# Brain–Immune Interactions as the Basis of Gulf War Illness: Clinical Assessment and Deployment Profile of 1990–1991 Gulf War Veterans in the Gulf War Illness Consortium (GWIC) Multisite Case-Control Study

**DOI:** 10.3390/brainsci11091132

**Published:** 2021-08-26

**Authors:** Lea Steele, Nancy Klimas, Maxine Krengel, Emily Quinn, Rosemary Toomey, Deborah Little, Maria Abreu, Kristina Aenlle, Ronald Killiany, Bang-Bon Koo, Patricia Janulewicz, Timothy Heeren, Allison N. Clark, Joy Ajama, Joanna Cirillo, Gerardo Buentello, Vanesa Lerma, Janet K. Coller, Kimberly Sullivan

**Affiliations:** 1 Veterans Health Research Program, Beth K. and Stuart C. Yudofsky Division of Neuropsychiatry, Department of Psychiatry and Behavioral Sciences, Baylor College of Medicine, Houston, TX 77030, USA; buentellojerry@prahs.com (G.B.); vanesa.lerma@bcm.edu (V.L.); 2 Institute for Neuroimmune Medicine, Dr. Kiran C. Patel College of Osteopathic Medicine, Nova Southeastern University, Fort Lauderdale, FL 33314, USA; nklimas@nova.edu (N.K.); mabreu1@nova.edu (M.A.); kaenlle@nova.edu (K.A.); 3 Department of Veterans Affairs, Miami VA Healthcare System, Miami, FL 33125, USA; 4 Department of Neurology, Boston University School of Medicine, Boston, MA 02118, USA; mhk@bu.edu; 5 Department of Biostatistics, Boston University School of Public Health, Boston, MA 02118, USA; eq@bu.edu (E.Q.); tch@bu.edu (T.H.); 6 Department of Psychological and Brain Sciences, Boston University College of Arts and Sciences, Boston, MA 02215, USA; toomey@bu.edu; 7 Department of Psychiatry and Behavioral Sciences, University of Texas Health Science Center at Houston, Houston, TX 77030, USA; Deborah.M.Little@uth.tmc.edu; 8 Department of Environmental Health, Boston University School of Public Health, Boston, MA 02118, USA; killiany@bu.edu (R.K.); bbkoo@bu.edu (B.-B.K.); paj@bu.edu (P.J.); ajamajoy@bu.edu (J.A.); jcirillo@bu.edu (J.C.); 9 H. Ben Taub Department of Physical Medicine and Rehabilitation, Baylor College of Medicine, Houston, TX 77030, USA; anclark@bcm.edu; 10 Discipline of Pharmacology, School of Biomedicine, University of Adelaide, Adelaide, SA 5005, Australia; janet.coller@adelaide.edu.au

**Keywords:** Gulf War illness, brain–immune interactions, military exposures, pesticides, traumatic brain injury, case-control study

## Abstract

The Boston University-based Gulf War Illness Consortium (GWIC) is a multidisciplinary initiative developed to provide detailed understanding of brain and immune alterations that underlie Gulf War illness (GWI), the persistent multisymptom disorder associated with military service in the 1990–1991 Gulf War. The core GWIC case-control clinical study conducted in-depth brain and immune evaluation of 269 Gulf War veterans (223 GWI cases, 46 controls) at three U.S. sites that included clinical assessments, brain imaging, neuropsychological testing, and analyses of a broad range of immune and immunogenetic parameters. GWI cases were similar to controls on most demographic, military, and deployment characteristics although on average were two years younger, with a higher proportion of enlisted personnel vs. officers. Results of physical evaluation and routine clinical lab tests were largely normal, with few differences between GWI cases and healthy controls. However, veterans with GWI scored significantly worse than controls on standardized assessments of general health, pain, fatigue, and sleep quality and had higher rates of diagnosed conditions that included hypertension, respiratory and sinus conditions, gastrointestinal conditions, and current or lifetime depression and post-traumatic stress disorder. Among multiple deployment experiences/exposures reported by veterans, multivariable logistic regression identified just two significant GWI risk factors: extended use of skin pesticides in theater (adjusted OR = 3.25, *p* = 0.005) and experiencing mild traumatic brain injury during deployment (OR = 7.39, *p* = 0.009). Gulf War experiences associated with intense stress or trauma (e.g., participation in ground combat) were not associated with GWI. Data and samples from the GWIC project are now stored in a repository for use by GWI researchers. Future reports will present detailed findings on brain structure and function, immune function, and association of neuroimmune measures with characteristics of GWI and Gulf War service.

## 1. Introduction

The 1990–1991 Persian Gulf War was among the most impressive military campaigns of the modern era. In response to Iraq’s military invasion of neighboring Kuwait in August 1990, U.S. and Coalition forces flooded into the region over a period of months. The active combat offensive, U.S. codenamed Operation Desert Storm, began with air strikes in January 1991 and ended with a ceasefire in February 1991 after just four days of ground combat [1]. But after the successful execution of the Gulf War, a substantial number of military personnel returned home with difficult health problems that were not explained by familiar medical or psychiatric diagnoses [2,3,4]. This condition, now known as Gulf War illness (GWI), remains a serious problem for Gulf War veterans 30 years after the war [5,6,7].

Relatively little was understood about the nature or causes of GWI in the early years after Desert Storm. As the years passed, a series of population studies identified a consistent profile of excess symptoms that affected up to one third of Gulf War veterans [8,9,10,11]. Multiple studies, using multivariable assessment methods, were also consistent in characterizing the most prominent GWI risk factors from among numerous stressors and potentially hazardous exposures that Gulf War troops encountered in theater [12,13,14,15,16,17]. In addition, clinical studies conducted in Gulf War veteran populations identified a series of neurological, immune and other pathobiological alterations that significantly distinguished GWI cases from healthy controls [18,19,20,21,22,23,24,25]. In parallel, studies using animal models to simulate the exposure experiences of Gulf War military personnel identified chronic and/or delayed neurological, inflammatory, and behavioral changes that were consistent with veterans’ chronic symptoms [26,27,28,29,30]. Taken together, preclinical, clinical, and population studies converged to indicate that the complex etiology and pathobiology of GWI involved persistent brain and inflammatory alterations likely triggered by a limited number of deployment exposures during the 1990–1991 Gulf War.

While these findings represented important progress for understanding GWI, there remained an urgent need for improved diagnosis and effective treatments for veterans who continued to suffer from this condition many years after the war. To address these objectives, the Office of Congressionally Directed Medical Research Programs (CDMRP) of the U.S. Department of Defense sponsored research consortia that enlisted scientific expertise in diverse disciplines to advance understanding, diagnosis and treatment of GWI. The Brain–Immune Interactions as the Basis of GWI: Gulf War Illness Consortium (GWIC) was developed as a multisite, interdisciplinary research program capable of integrating and building on GWI findings in multiple fields.

Headquartered at Boston University, the GWIC included multiple sites and coordinated projects to determine the specific neurological, inflammatory, and neuroimmune processes that underlie the symptoms of GWI, with the central objective of identifying GWI biomarkers and treatments. The ten GWIC participating institutions include five sites that conducted studies of veterans who served in the Gulf War (Boston University, Miami VA, Nova Southeastern University, Baylor College of Medicine, University of Adelaide) and five sites that conducted animal and in vitro GWI studies (U.S. Centers for Disease Control and Prevention (CDC), National Institutes of Health, University of Colorado, Drexel University, Temple University). A central feature of the GWIC has been coordination of clinical studies of Gulf War veterans with animal studies that characterize persistent effects of Gulf War exposures on the brain and on neuroimmune processes. Animal models are also used to test therapeutic compounds that counteract these effects and have the potential to provide beneficial GWI treatments. Data and samples from the GWIC project are now stored in a repository for use by GWI researchers.

The present report provides an overview of the core GWIC clinical project, a large three-site GWI case-control study conducted in Boston, Miami, and Houston. This study provided multifaceted evaluation of brain and immune parameters in Gulf War veterans that included detailed assessments of immune function and immunogenetic factors, magnetic resonance imaging (MRI) assessment of brain structure and function, neuropsychological testing, and clinical assessment of general health and psychiatric status. Here we describe study methods and data collected for the three-site project, compare general health measures between GWI cases and controls, and identify deployment experiences and exposures found to be significantly associated with GWI case status. Future reports will provide in-depth results from GWIC brain imaging, brain function, and laboratory assessments to determine the specific neurological, cognitive, immune and genetic parameters that underlie the symptoms of GWI.

## 2. Materials and Methods

### 2.1. Study Recruitment, Screening, and Participation

Data were collected for the GWIC case-control study between 2015 and 2020 at three clinical study sites: Boston University, the Miami Department of Veterans Affairs Medical Center (VAMC), and Baylor College of Medicine in Houston. Project recruitment was conducted through extensive outreach efforts to inform Gulf War veterans about the study via veterans groups and meetings, media articles, social media, and veteran referrals. After initial contact with the research team to obtain study information, interested veterans were invited to participate in a structured telephone interview to determine their study eligibility. Consenting veterans answered questions about their Gulf War military deployment, medical history, and current health. Those who met eligibility criteria were provided additional study information and, if interested, were invited to participate in the full study, which required a 1-day study visit. At the study site, veterans received detailed study information and provided informed consent prior to participating in the series of research evaluations and testing included in the study protocol, as described below. Study protocol and informed consent documents were approved by institutional review boards at Boston University, Miami VAMC, and Baylor College of Medicine and reviewed by the U.S. Army Medical Research and Development Command’s Office of Human Research Protections.

### 2.2. Eligibility Criteria and GWI Case Definition

Veterans were eligible for the study if they had deployed to the Gulf War Theater of Operations for any period between August 1990 and July 1991, were able to provide informed consent, and had not previously been diagnosed with any conditions designated as exclusionary for purposes of the GWIC project, as noted below. Primary GWI case/control status was determined using the Kansas GWI case definition criteria [10]. Additional data were collected to determine if veterans also met CDC criteria for chronic multisymptom illness (CMI), as defined by Fukuda et al. [8].

Briefly, the Kansas GWI case definition inclusionary criteria require that veterans endorse multiple and/or moderate to severe symptoms as problems that had persisted or recurred over six months in at least three of six defined symptom domains. Defined symptom domains include: (1) fatigue/sleep problems, (2) pain symptoms, (3) neurological/cognitive/mood symptoms, (4) gastrointestinal symptoms, (5) respiratory symptoms, and (6) skin symptoms. The Kansas criteria also exclude as GWI cases any veterans diagnosed with conditions that could account for their chronic symptoms or interfere with their ability to accurately report them (e.g., severe psychiatric disorders). Notably, Kansas GWI criteria do not exclude subjects with other unexplained symptom-defined conditions, such as fibromyalgia (FM), chronic fatigue syndrome/myalgic encephalomyelitis (CFS), or irritable bowel syndrome (IBS) [10].

Consistent with the Kansas GWI criteria, lead investigators at the three study sites (KS, NK, LS) established a list of medical and psychiatric conditions that were pre-specified as exclusionary for the GWIC study. The GWIC exclusionary criteria also designated time frame parameters to allow for prior conditions that had resolved or were adequately managed, and so could not account for veterans’ symptoms at the time of the study.

Exclusionary conditions. The GWIC study eligibility criteria excluded veterans who had ever been diagnosed by a physician with multiple sclerosis, lupus, rheumatoid arthritis, stroke, Parkinson’s disease, amyotrophic lateral sclerosis (ALS, or Lou Gehrig’s disease), Alzheimer’s disease, bipolar disorder, or schizophrenia. Criteria also excluded veterans if they had previously been diagnosed with any of the following conditions and there was any indication the condition was still active in the five years before the study: seizure disorder, heart disease (high blood pressure or high cholesterol not exclusionary), kidney disease, liver disease, cancer (except non-melanoma skin cancer, which was not exclusionary). For example, veterans who had a prior cancer diagnosis but had been told by their providers that they had been cancer–free for at least 5 years were not excluded from the study. Veterans diagnosed with diabetes were included only if their blood sugar had been well controlled for at least 2 years but were otherwise excluded. Veterans were also excluded if they had a chronic infectious disease lasting six months or longer or were recovering from a serious injury that could account for their symptoms. In addition, veterans were excluded if they had been hospitalized for post-traumatic stress disorder, depression, or alcohol or drug dependence in the previous 5 years. GWI exclusionary criteria established for the GWIC study are summarized in Supplemental Figure S1.

Exclusionary criteria were applied to all screened participants, regardless of their likely case status, to ensure a comparison group of healthy controls and minimize the potential for any case-control differences identified by the study to be the result of conditions other than GWI.

### 2.3. Data Collection

#### 2.3.1. Screening Interview

Veterans who consented to the screening interview were asked if they had deployed to the Persian Gulf region for any period between August 1990 and July 1991, then responded to a series of questions about symptoms associated with Kansas GWI and CMI criteria. For symptoms identified as persistent problems over the prior 6 months, veterans were asked to rate the problem as mild, moderate or severe. Veterans were also asked about their medical history, including hospitalizations and conditions diagnosed by a healthcare provider that could potentially affect study eligibility. Eligible veterans were provided additional study information and invited to schedule an in-person study visit.

#### 2.3.2. GWIC Study Visit

Upon arriving at the study site the morning of their appointment, veterans were given detailed study information, and any questions were discussed prior to obtaining informed consent. Consenting veterans provided fasting blood samples and initial saliva samples, followed by a brief physical evaluation to obtain data on height, weight, vital signs (including supine and standing blood pressure) and fibromyalgia tender points. Participants were then administered a neuropsychological testing battery that included tests of executive system functioning, attention, motor function, visuospatial function, memory, mood, and motivation. Veterans also received a clinical psychiatric interview that included the Clinician Administered PTSD Scale (CAPS) [31] and the Structured Clinical Interview for DSM-V (SCID) [32] to identify exclusionary diagnoses (bipolar disorder, schizophrenia) and/or comorbid psychiatric diagnoses (major depression, anxiety disorder, dysthymia, post-traumatic stress disorder (PTSD)). In addition, at the Boston and Houston sites, study participants with no safety contraindications received a magnetic resonance imaging (MRI) scan of the brain. The neuroimaging battery included a T1-weighted magnetization-prepared rapid acquisition (MPRAGE) sequence, multi-component T-2 scan, diffusion tensor imaging (DTI), fMRI, pCASL sequence, and a High Angular Resolution Diffusion Imaging (HARDI DTI) scan [33,34].

#### 2.3.3. Standardized Health Assessments and Study Questionnaires

During the course of their study visit, veterans were administered a series of standardized health assessments and completed additional questionnaires online during or after their study visit. Health assessments included the Veterans SF36 [35], McGill Pain Questionnaire [36], Pain Visual Analog Scale, Multidimensional Fatigue Inventory [37], Pittsburgh Sleep Quality Index [38], and the Structured Neurotoxicant Assessment Checklist (SNAC) [39]. Veterans also completed the Kansas Gulf War and Health Questionnaire [15], which queries a broad range of experiences and exposures specifically associated with Gulf War service and the duration of each exposure. In addition, veterans were asked if they had experienced one or more mild traumatic brain injuries (mTBI) before, during, or after Gulf War deployment, and the estimated number of mTBIs during each period. For purposes of the study, mTBI was defined according to American Academy of Neurology guidelines [40] as an impact to the head that causes symptoms for any amount of time (i.e., seconds or longer)—symptoms that may have included sensitivity to light or noise, headache, dizziness, balance problems, nausea, vomiting, trouble sleeping, fatigue, confusion, difficulty remembering, difficulty concentrating, or loss of consciousness.

#### 2.3.4. Testing of Collected Samples

Blood analyses included a battery of standard clinical diagnostic tests (complete blood count, basic metabolic panel, thyroid panel, rheumatoid factor, antinuclear antibody). In addition, participants’ de-identified blood and saliva samples were shipped by overnight courier to the project’s central research laboratory and repository site, the E.M. Papper Immunology Laboratory at Nova Southeastern University, where extensive research testing was conducted. Research analyses included multiplex evaluation of a comprehensive panel of cytokines and chemokines in the blood and nanostring analyses of mRNA and miRNA of proteins associated with toll like receptor functioning and glial activation. In addition, cortisol levels were tested in saliva samples collected at regular intervals throughout the study visit. Saliva samples were also shipped to the GWIC collaborating genetic laboratory at the University of Adelaide to test for genetic markers associated with variability in immune and proinflammatory processes.

### 2.4. Data Management and Analyses

Data collected at the three clinical study sites, identified only by subjects’ study identification numbers, were securely submitted to Boston University’s Biostatistics and Epidemiology Data Analytics Center (BEDAC) for data consolidation, management, and analyses. Additional analyses were conducted by project investigators at individual study sites, to address specific research questions.

Data analyses for the current report involve bivariate and multivariable comparisons between GWI cases and controls using standard analytic methods. This included chi- square tests to evaluate case/control comparisons associated with categorical variables. Comparison of categorical outcomes for which any expected cell size was <5 utilized Fisher’s exact test to determine *p*-values. Mean values of continuous variables were compared using T tests, according to observed distributions of individual variables. Significance was assessed by *p*-values determined using pooled variances, when equal for GWI cases and controls, and the Satterthwaite method [41,42] when variances were not equal. Prevalence odds ratios and 95% confidence intervals were used to estimate the magnitude of association of GWI with veteran-reported experiences/exposures during Gulf War deployment—both unadjusted (bivariate) associations and adjusted (multivariable) associations.

Multivariable logistic regression was used to identify independent associations of Gulf War experiences and exposures with GWI, adjusted for effects of covariates as well as potential confounding effects of concurrent exposures. The multistep modeling approach first used results of bivariate analyses to test all significant associations of GWI with deployment experiences/exposures in a single model. Final models retained individual exposures significant at *p* < 0.05, as well as prominent variables (age, rank, PTSD) that differed between GWI cases and controls in initial bivariate analyses. All analyses were conducted using SAS/STAT statistical analysis software, version 9.4 [43].

## 3. Results

### 3.1. Study Sample

Overall, 703 veterans were screened for study eligibility at the three GWIC sites. Twenty-eight veterans indicated they had not served in theater for any period between August 1990 and July 1991 and were not further evaluated. Of the 675 remaining, 436 (65%) were identified as study eligible, and 239 were not eligible due to previous diagnoses of one or more exclusionary conditions. Four hundred eleven screened veterans were invited to participate in the full study, and 271 (66%) completed study appointments. Two veterans who completed study visits were subsequently excluded from the final sample based on additional health information obtained during study evaluations. The final GWIC study sample therefore included 269 Gulf War veterans: 223 GWI cases and 46 veteran controls. This included 147 veterans evaluated at the Boston site, 50 evaluated at the Miami site, and 72 evaluated at the Houston site.

Demographic, military, and deployment characteristics of the study sample are provided in Table 1. Overall, GWI cases were similar demographically to veteran controls but included a somewhat higher proportion of women (17% GWI cases vs. 9% controls, *p* = 0.16). Age group distributions were similar by decade, although the mean age of GWI cases was about two years younger than veteran controls (*p* = 0.04). Veterans’ military characteristics at the time of the Gulf War were also similar, with one exception. The large majority of GWI cases (90%) had served in the enlisted ranks during the Gulf War, compared to only 70% of controls (OR =3.84, *p* = 0.0003). However, there were no case/control differences by military branch, service component, the time period veterans spent in theater, or the duration of veterans’ deployment. Overall, 89% of all veterans in the sample had been in theater during the two months of active combat, January–February 1991. The remaining 11% either left the region during Operation Desert Shield, before the onset of air strikes, or arrived in theater after the cease-fire was declared in late February 1991.

### 3.2. General Health, Medical History, and Standardized Health Assessments

General health characteristics of GWI cases and controls are compared in Table 2. As shown, similar proportions of cases and controls recalled being in good to excellent health prior to Gulf War deployment, and were regular smokers both during the Gulf War and at the time of the study. As expected, however, GWI cases indicated worse overall health than controls at the time of the study. This disparity was reflected in significant case/control differences in veterans’ medical history, and by standardized health and psychiatric assessments conducted at the time of the study.

As described, veterans who were previously diagnosed with any designated exclusionary conditions were not eligible for the study. As shown in Table 2, however, a significantly greater proportion of GWI cases than controls reported they had been diagnosed by a physician with a number of nonexclusionary medical conditions including hypertension, allergies or sinus problems, gastrointestinal conditions and two chronic multisymptom conditions: irritable bowel syndrome (IBS) and chronic fatigue syndrome (CFS). Structured psychiatric evaluations conducted for the study also indicated that a significantly greater proportion of GWI cases than controls met criteria for major depression (current or lifetime), anxiety disorder, and post-traumatic stress disorder (current or lifetime).

In addition, GWI cases scored significantly more poorly than controls on each of the standardized health assessments administered for the study. This included evaluations of general health and quality of life, pain, fatigue, and sleep quality. For example, whereas mean values for veteran controls on both the physical component (PCS) and mental component (MCS) summary scores of the Veterans SF36 were near the normal value of 50, GWI cases scored significantly worse on both the PCS (mean = 35, *p* < 0.001 vs. controls) and MCS (mean = 41, *p* < 0.001 vs. controls). Gulf War illness cases also reported significantly higher levels of pain on both their best and worst days and tested positive for a significantly greater number of fibromyalgia (FM) tender points than controls. Twenty-eight percent of GWI cases had 11 or more positive tender points out of 18 tested, consistent with 1990 diagnostic criteria for fibromyalgia [44], vs. only 2% of controls (*p* < 0.001).

Despite the significant degree of poor health indicated by both medical history and health assessments, veterans with GWI, overall, had mostly normal health indicators on standard physical evaluation and clinical diagnostic tests conducted for the study. As detailed in Table 2, no case/control differences were observed in relation to veterans’ vital signs, height, weight, or body mass index. Few veterans exhibited possible evidence of postural orthostatic hypotension when comparing diastolic and systolic blood pressure moving from laying down to a standing position, with no significant differences between GWI cases and controls.

### 3.3. Blood Testing

Clinical reference lab testing of fasting blood samples taken the morning of the study identified only a limited number of differences between GWI cases and controls. Among basic metabolic panel (BMP) tests, GWI cases differed significantly from controls on mean levels of CO_2_, glucose, and bilirubin. A greater proportion of GWI cases than controls had elevated fasting blood glucose levels (13% vs. 2%, *p* = 0.04), while a greater proportion of controls had higher-than-normal CO_2_ and bilirubin levels. No significant case/control differences were associated with lipid panel tests, thyroid stimulating hormone, antinuclear antibodies, or rheumatoid factor. Of note, 38–42% of both cases and controls had elevated total and LDL serum cholesterol levels.

Complete blood count (CBC) testing also identified a limited number of case/control differences. These included significant differences in mean white blood cell (WBC) counts (*p* = 0.007), with more controls than cases having WBC counts below the reference range. Controls also had significantly greater mean percent monocytes than cases (*p* = 0.04), while cases had a larger mean red cell distribution width (RDW) than controls (*p* = 0.03).

### 3.4. Symptom Profiles: GWI Cases and Controls

Table 3 identifies the proportion of GWI cases and controls who endorsed each of the Kansas GWI criteria symptoms as persisting for six months or longer. Kansas GWI symptom criteria require two mild or one moderate-severe chronic symptom in at least 3 of 6 domains, a minimum of 3–6 symptoms. For the GWIC sample, however, each of the 29 individual GWI symptoms were endorsed by significantly more GWI cases than controls. Both GWI cases and controls endorsed symptoms at substantially higher frequencies than was typically observed in early Gulf War veteran studies. For example, over 90% of GWI cases endorsed fatigue, pain, and cognitive chronic symptoms, while 50% of controls endorsed chronic joint pain and 41% had sleeping difficulties. Still, a significantly greater proportion of GWI cases than controls endorsed multiple or moderate-severe symptoms in each of the six defined symptom domains.

We also assessed chronic symptoms associated with the CMI case criteria [8]. Nearly all GWI cases in our study (*n* = 221, 99%) met criteria for CMI, and half of veteran controls (*n* = 23, 50%) also met CMI criteria.

### 3.5. Association of Deployment Experiences and Exposures with GWI

Veterans reported a broad range of experiences and exposures during Gulf War deployment. Table 4 compares the overall proportion of GWI cases and controls who reported ever having each experience/exposure in theater, as well as the proportion who experienced each item for seven days or longer. Initial bivariate comparisons between GWI cases and controls suggested that 14 of the 23 Gulf War experiences/exposures queried were potentially associated with GWI, with unadjusted OR point estimates ranging from 1.99–8.33 (*p* < 0.05). A high degree of correlation was observed among reported deployment exposures, however, suggesting the potential for confounding error when evaluating exposure-GWI associations individually.

We therefore utilized logistic regression to identify independent associations of deployment experiences and exposures with GWI. After controlling for veterans’ age, rank, PTSD status, and significant deployment experiences/exposures, adjusted models identified only two significant deployment risk factors for GWI. These included: (1) extended (≥7 days) use of cream or spray pesticides on the skin (adjusted OR = 3.25, *p* = 0.005) and (2) experiencing one of more mTBIs during deployment (adjusted OR = 7.39, *p* = 0.009).

In contrast, veterans who reported having one or more mTBIs prior to Gulf War deployment (38% cases vs. 35% controls, *p* = 0.71) or after the war (31% cases vs. 26% controls, *p* = 0.50) were not at increased risk for GWI. Further, no interactions were observed between deployment mTBI and pesticide use or other potential neurotoxicant exposures (e.g., hearing chemical alarms, use of pyridostigmine bromide) in relation to the risk for GWI.

Stressful deployment experiences were not identified as risk factors for GWI, although several were significantly associated with PTSD (not shown). For example, participation in ground combat was not a significant risk factor for GWI in our sample but was significantly associated with PTSD (OR = 3.55, *p* < 0.001).

## 4. Discussion

The GWIC case-control study, the core clinical project of the Boston University-based GWI research consortium, provided in-depth assessment of brain and immune function of 1990–1991 Gulf War veterans at three U.S. sites. Here we describe the general health of GWIC participants, results of clinical evaluation and testing of GWIC cases and controls, and significant GWI risk factors among veteran-reported wartime experiences and exposures.

In the three-site sample, GWI cases were generally similar to controls in relation to demographic, military, and deployment characteristics, although GWI cases were 2 years younger on average, and included a significantly higher proportion of veterans who had served in the enlisted ranks (vs. officers) during the war. This is similar to previous Gulf War veteran studies, where one of the most consistent findings has been a higher prevalence of GWI in enlisted personnel compared to officers [10,11,13,14]. This potentially reflects differences between officers and enlisted personnel in relation to Gulf War deployment activities and exposures. Such differences also parallel health differences observed in nonmilitary populations, for example, patterns of greater morbidity among civil servants serving in lower vs. higher ranks [45].

In addition to GWI symptoms, GWI cases had multiple indicators of poor health that distinguished them from controls. Veterans with GWI scored significantly worse on standardized assessments of general health status, pain, fatigue, and sleep quality compared to controls. They also reported a higher prevalence of physician-diagnosed hypertension, allergies and sinus problems, irritable bowel syndrome, other gastrointestinal disorders, and chronic fatigue syndrome, and were more likely to have current or lifetime PTSD and major depression.

Despite the degree of ill health associated with GWI, results of standard physical evaluation and clinical diagnostic tests were mostly normal, with only limited differences that distinguished GWI cases from healthy controls. This is consistent with earlier Gulf War veteran reports [8,46,47] and exemplifies a longstanding challenge associated with GWI for veterans and their healthcare providers. Symptoms, by definition, are patients’ own experiences as opposed to externally “objective” measures of disease and have thus far been the only consistent marker of GWI in affected veterans. For many years, the lack of diagnosable abnormalities on physical exam and routine clinical lab tests commonly led to provider assumptions that nothing was wrong with veterans who reported chronic GWI symptoms, or that their health problems were the result of deployment stress and/or psychiatric in nature [48,49,50].

Such assumptions have not been supported by the large body of Gulf War population and clinical studies that have routinely indicated that GWI is not the result of serving in combat or other wartime stressors. Rather, the most consistently identified GWI risk factors have been neurotoxicant exposures during Gulf War deployment [11,12,13,14,15,16,17,51]. For the current study, GWIC participants reported a broad range of experiences and exposures during their wartime service. But only two—extended use of skin pesticides and having one or more mild traumatic brain injuries in theater—were significant risk factors for GWI. Our finding of extended personal pesticide use as a prominent GWI risk factor was consistent with previous studies [11,12,13,14,15,16,17,24]. The lack of association of GWI with serving in combat and other deployment stressors was also consistent with previous studies [11,12,13,14,15]. However, unlike previous studies, use of pyridostigmine bromide (nerve agent pyridostigmine pretreatment, or NAPP) pills was not identified as a risk factor for GWI in our study. The wide use of NAPP pills as a protective measure against potential deadly effects of chemical nerve agents was unique to the 1990–1991 Gulf War and was reported by a high proportion of both GWI cases (82%) and controls (67%) in our sample.

Having one or more mild TBIs during Gulf War deployment was also identified as a significant GWI risk factor in the current study, although mTBIs before or after the Gulf War were not. Brain injuries are commonly recognized as health concerns for veterans of post 9/11 deployments but have seldom been evaluated in relation to chronic health outcomes in 1990–1991 Gulf War veterans. A limited number of Gulf War veteran studies have previously assessed mTBIs in relation to GWI, with varying results. In the Fort Devens cohort, the prevalence of GWI was elevated among veterans who reported a history of three or more mTBIs [52]. TBIs during deployment were infrequently reported in a VA study of 202 Gulf War veterans [53]. There, a history of TBIs overall was associated with symptomatic illness, broadly defined, but not with more narrowly-defined GWI. In addition, two prior studies have reported on effects of mTBI in subsets of the full GWIC case-control sample evaluated here. The first reported a significant association of mTBIs sustained in theater with GWI [54]. The second identified focal microstructural differences on high order diffusion MRI brain scans among veterans with GWI who reported having mTBIs during deployment, compared to veterans with no mTBI [34].

### 4.1. Symptom Reporting and GWI Case Definition

Primary GWI case status for the GWIC study was based on the Kansas GWI case criteria [10], with secondary assessment of CMI criteria [8]. Both case definitions were developed in the first decade after the Gulf War and based on the types and pattern of symptoms reported by Gulf War veterans at that time, with Kansas criteria also providing guidelines for excluding veterans with diagnoses that potentially explain their symptoms. By design, GWI cases in our study endorsed significantly more symptoms than controls. However, the frequency of symptoms reported by GWI cases was higher than generally observed in early GWI studies and substantially greater than the symptom burden required to meet Kansas GWI case criteria. This is consistent with other more recent studies of Gulf War veterans that have generally indicated that, over the years, 1990–1991 Gulf War veterans have reported an increasing burden both of chronic symptoms and of diagnosed health conditions [5,6,7,55,56]. In the current study, this increased symptom burden was also observed in controls, half of whom met CMI criteria.

More than 20 years after the CMI and Kansas GWI case definitions were developed, time and age-associated changes in both the symptom profile and diagnosed conditions affecting Gulf War veterans suggest the likelihood of reduced specificity for both case definitions. This, in turn, would be expected to reduce their utility for accurately characterizing GWI cases for research and other purposes. Increased levels of morbidity observed in this and other studies supports the need for evidence-based revisions to existing GWI case definitions [6,57,58,59]. A primary consideration is that revised GWI criteria more accurately reflect present day symptoms and diagnosed health conditions associated with service in the 1990–1991 Gulf War in order to optimally distinguish GWI cases from noncases.

### 4.2. Strengths and Limitations

The GWIC case-control study has several strengths and limitations to consider in assessing research results. Important strengths are the study size and rigorous characterization of veterans for the project, which included a diverse sample of 1990–1991 Gulf War veterans from different regions in the country. To our knowledge, the GWIC multisite project (n = 269) represents the most comprehensive evaluation of clinical, neurological and immune measures of 1990–1991 Gulf War veterans to date and is one of the largest case-control studies conducted in this population. However, the sample included fewer controls than originally targeted for the study, which may prove to be a key limitation in addressing some study questions. In addition, the GWIC study sample was not randomly selected from a defined population, so the extent to which cases and controls are representative of the larger Gulf War veteran population is uncertain.

This initial report from the full GWIC case-control study sample provides an overview of research assessments and general health comparisons between GWI cases and controls. Future papers will report on results of neuroimaging, neurocognitive, immune, and genetic testing from Gulf War veterans evaluated at the three GWIC clinical sites, including the degree to which identified health outcomes differ in GWI subgroups and are associated with exposures during the Gulf War.

## 5. Conclusions

Limited findings on routine clinical assessment of ill Gulf War veterans underscores the importance of applying multidisciplinary, state of the art research to accelerate progress in addressing GWI.

Improved understanding of brain and immune GWI pathobiology provided by the GWIC and related projects is essential for identifying effective treatments and valid diagnostic tests for the complex of serious health problems that continue to affect Gulf War veterans, 30 years after their service in Operation Desert Storm.

## Figures and Tables

**Table 1:** Demographic, military, and deployment characteristics of GWI cases and controls.

	GWI Cases (*n* = 223)	GW Veteran Controls (*n* = 46)	Test Statistic	*p* Value
**Sex**				
Female	17%	9%	2.01 ^1^	*p* = 0.16
Male	83%	91%		
**Age**				
43–49	44%	30%	3.47 ^1^	*p* = 0.18
50–59	45%	52%		
60+	11%	17%		
Mean age (years)	52.2	54.2	2.07 ^2^	*p* = 0.04
**Race**				
Black/African American	13%	11%	0.18 ^1^	*p* = 0.92
White/Caucasian	79%	80%		
Other/Mixed	8%	9%		
**Hispanic ethnicity**	9%	4%	na ^3^	*p* = 0.39
**Highest Education Level**			0.61 ^1^	*p* = 0.89
High school or GED	6%	9%		
Some college or training after high school	49%	46%		
4 year degree	20%	20%		
Advanced degree	24%	26%		
**Rank in 1990**				
Enlisted	90%	70%	13.01 ^1^	*p* < 0.001
Officer	10%	30%		
**Branch of Service in 1990**				
Army	65%	72%	2.47 ^1^	*p* = 0.48
Navy	12%	15%		
Air Force	7%	4%		
Marines	16%	9%		
**Service Component in 1990**				
Regular (Active Component)	78%	76%	0.67 ^1^	*p* = 0.72
Reserves	17%	15%		
National Guard	6%	9%		
**Gulf War Deployment: Service Period in Theater**				
Present Jan-Feb 1991 and departed by May 1991	71%	72%	0.91 ^1^	*p* = 0.63
Present Jan-Feb 1991 and departed after May 1991	17%	21%		
Departed prior to Jan 1991 or arrived after Feb 1991	11%	7%		
Mean number of months in theater	6.5	6.7	0.45 ^2^	*p* = 0.65

Abbreviations: GW = Gulf War; GWI = Gulf War illness; na = not applicable. Statistical tests: ^1^ chi-square; ^2^ T test; ^3^ Fisher’s exact test.

**Table 2:** General health characteristics of GWI cases and controls.

	GWI Cases (*n* = 223)	GW Veteran Controls (*n* = 46)	Test Statistic	*p* Value
**Veteran-reported health status prior to Gulf War deployment**				
Excellent	92%	87%	1.24 ^1^	*p* = 0.26
Good	8%	13%		
**Veteran-reported health status at time of study**				
Excellent	2%	20%	71.5 ^1^	*p* < 0.001
Good	14%	50%		
Fair	37%	30%		
Poor	47%	0		
**Regular smoker**				
During Gulf War deployment	27%	24%	0.24 ^1^	*p* = 0.62
At time of study	10%	7%	na ^3^	*p* = 0.59
**Medical History: Physician-diagnosed conditions** (not exclusionary for GWI)				
Hypertension	45%	28%	4.55 ^1^	*p* = 0.03
Respiratory allergies/sinus problems	42%	7%	20.46 ^1^	*p* < 0.001
Irritable bowel syndrome	31%	7%	11.62 ^1^	*p* < 0.001
Other gastrointestinal diagnosis	31%	9%	9.35 ^1^	*p* < 0.01
Chronic fatigue syndrome	23%	2%	10.71 ^1^	*p* < 0.001
Asthma	14%	4%	3.02 ^1^	*p* = 0.08
Chemical sensitivity	7%	0	na ^3^	*p* = 0.14
**Psychiatric Diagnoses: Evaluated at time of study**				
Major Depression: Current or lifetime	44%	24%	5.54 ^1^	*p* = 0.02
Dysthymia: Current or lifetime	7%	3%	1.00 ^1^	*p* = 0.32
Anxiety Disorder: Current	14%	0	na ^3^	*p* = 0.01
Post-traumatic Stress Disorder: Current or lifetime	56%	23%	15.43 ^1^	*p* < 0.001
**Standardized Health Assessments: Evaluated at time of study**				
**General Health**				
Veterans SF-36 mean Physical Component Summary Score	35	50	12.86 ^2^	*p* < 0.001
Veterans SF-36 mean Mental Component Summary Score	41	51	4.99 ^2^	*p* < 0.001
**Pain**				
Magill Pain Inventory mean score (0–78)	32.5	16.4	−7.38 ^2^	*p* < 0.001
Average pain level on best days (visual analog scale, 0–100)	26.6	10.1	−6.91 ^2^	*p* < 0.001
Average pain level on worst days (visual analog scale, 0–100)	72.4	41.9	−7.38 ^2^	*p* < 0.001
Fibromyalgia tender point exam				
Mean number of positive FM tender points (of 18)	6	1	−8.19 ^2^	*p* < 0.001
Veterans with 11+ tender points	28%	2%	13.97 ^1^	*p* < 0.001
**Fatigue**			−8.19 ^2^	
Multidimensional Fatigue Inventory (MFI) mean score (0–100)	64.6	38.3		*p* < 0.001
**Sleep**				
Pittsburgh Sleep Quality Index mean score (0–21)	13.0	7.5	−9.12 ^2^	*p* < 0.001
**Physical evaluation at time of study**				
Oral temperature (mean ^o^F)	97.8	97.9	0.292 ^2^	*p* = 0.77
Resting pulse (mean beats/minute)	70	68	−0.982 ^2^	*p* = 0.33
Height (mean inches)	69	70	1.482 ^2^	*p* = 0.14
Mean weight (pounds)	217	216	−0.292 ^2^	*p* = 0.77
Body mass index (mean)	32	31	−1.002 ^2^	*p* = 0.32
Supine blood pressure (mean systolic/mean diastolic)	134/82	134/79	-	ns
Standing blood pressure (mean systolic/mean diastolic)	132/88	131/86	-	ns
Diastolic drop of 10 or more points after standing	4%	2%	na ^3^	*p* = 1.00
Systolic drop of 20 or more points after standing	6%	2%	na ^3^	*p* = 0.48

Abbreviations: GW = Gulf War; GWI = Gulf War illness; FM = fibromyalgia; na = not applicable; ns = not statistically significant. Statistical tests: ^1^ chi-square; ^2^ T test; ^3^ Fisher’s exact test.

**Table 3:** Proportion of GWI cases and controls endorsing chronic symptoms.

Symptoms Identified as Persistent or Recurring Problems over the Previous 6 Months	GWI Cases ^1^ (*n* = 223)	GW Veteran Controls (*n* = 46)
**Fatigue/Sleep Domain**		
Not feeling rested after sleep	95%	35%
Fatigue	93%	22%
Problems getting to sleep or staying asleep	90%	41%
Feel unwell after physical exercise or exertion	79%	11%
*Multiple or moderate-severe symptoms*	99%	30%
**Pain Domain**		
Joint pain	94%	50%
Muscle pain	78%	11%
Body pain—hurt all over	70%	4%
*Multiple or moderate-severe symptoms*	92%	20%
**Neurologic/Cognitive/Mood Domain**		
Problems remembering recent information	91%	39%
Difficulty concentrating	90%	37%
Trouble finding words when speaking	83%	35%
Feeling irritable or having angry outbursts	80%	28%
Headaches	75%	13%
Feeling down or depressed	75%	33%
Numbness or tingling in extremities	71%	17%
Eyes sensitive to light	70%	20%
Feeling dizzy, lightheaded, or faint	65%	15%
Low tolerance for heat or cold	65%	9%
Night sweats	63%	11%
Symptomatic response to chemicals, odors	58%	9%
Blurred or double vision	55%	11%
Tremors or shaking	47%	4%
*Multiple or moderate-severe symptoms*	99%	57%
**Gastrointestinal Domain**		
Nausea or upset stomach	66%	4%
Diarrhea	64%	4%
Abdominal pain or cramping	61%	2%
*Multiple or moderate-severe symptoms*	72%	4%
**Respiratory Domain**		
Difficulty breathing or catching breath	63%	11%
Persistent cough when don’t have a cold	53%	11%
Wheezing in chest	37%	4%
*Multiple or moderate-severe symptoms*	61%	2%
**Skin Domain**		
Skin rashes	52%	11%
Other skin problems	35%	2%
*Multiple or moderate-severe symptoms*	41%	2%
Mean number of GWI symptom domains for which multiple or moderate-severe symptoms were endorsed	4.7	1.1

Note: ^1^ Frequency of all individual symptoms and GWI symptom domains significantly greater in GWI cases vs. controls, *p* < 0.001. Abbreviations: GW = Gulf War; GWI = Gulf War illness.

**Table 4:** Association of GWI case status with Gulf War deployment experiences and exposures.

Deployment Experiences/Exposures	% Exposed		OR (95% CI) (Unadjusted)	OR (95% CI) (Adjusted) ^1^
	GWI Cases (*n* = 223)	GW Controls (*n* = 46)		
Regular smoker during deployment	27%	24%	1.20 (0.57–2.52)	1.14 (0.45–2.88)
Saw smoke from oil well fires				
Ever	87%	83%	1.41 (0.60–3.32)	0.74 (0.26–2.09)
≥7 days	66%	57%	1.47 (0.77–2.80)	0.92 (0.41–2.06)
Heard chemical alarms sounded				
Ever	86%	72%	2.43 (1.15–5.14) *	0.67 (0.25–1.80)
≥7 days	50%	28%	2.56 (1.28–5.14) *	1.36 (0.58–3.16)
Within 1 mile of exploding SCUD missile				
Ever	50%	33%	2.05 (1.05–4.01) *	1.24 (0.54–2.86)
≥7 days	15%	7%	2.60 (0.76–8.87)	2.10 (0.40–11.14)
Directly involved in ground combat				
Ever	45%	33%	1.70 (0.87–3.33)	0.69 (0.29–1.64)
≥7 days	21%	15%	1.47 (0.62–3.52)	0.44 (0.15–1.32)
Directly involved in air combat				
Ever	10%	7%	1.56 (0.44–5.47)	1.38 (0.30–6.38)
Saw U.S. troops badly wounded or killed				
Ever	54%	37%	2.00 (1.04–3.85) *	0.79 (0.34–1.81)
≥7 days	22%	13%	1.87 (0.75–4.67)	1.01 (0.33–3.10)
Saw Iraqis badly wounded or killed				
Ever	72%	57%	1.99 (1.03–3.82) *	0.73 (0.31–1.70)
≥7 days	33%	26%	1.37 (0.67–2.80)	0.80 (0.33–1.96)
Contact with prisoners of war				
Ever	59%	46%	1.69 (0.89–3.20)	0.99 (0.44–2.22)
≥7 days	32%	24%	1.47 (0.71–3.07)	1.29 (0.50–3.32)
Saw dead animals				
Ever	72%	59%	1.78 (0.92–3.43)	0.76 (0.31–1.83)
≥7 days	32%	26%	1.34 (0.65–2.74)	0.39 (0.15–1.06)
Saw destroyed enemy vehicles				
Ever	85%	72%	2.25 (1.07–4.74) *	0.95 (0.36–2.50)
≥7 days	57%	39%	2.04 (1.06–3.91) *	0.68 (0.29–1.60)
Contact with destroyed enemy vehicles				
Ever	71%	46%	2.87 (1.50–5.50) *	1.45 (0.61–3.43)
≥7 days	40%	24%	2.12 (1.02–4.40) *	0.91 (0.36–2.27)
Contact with American vehicles hit by friendly fire				
Ever	37%	28%	1.47 (0.73–2.97)	0.71 (0.28–1.82)
≥7 days	15%	17%	0.86 (0.37–2.01)	0.32 (0.10–1.03)
Used pesticides cream/spray on skin				
Ever	74%	43%	3.69 (1.91–7.13) **	1.87 (0.87–4.02)
≥7 days	65%	26%	5.18 (2.53–10.59) **	3.25 (1.44–7.34) *
Wore uniform treated with pesticides				
Ever	53%	35%	2.16 (1.11–4.19) *	0.92 (0.36–2.40)
≥7 days	47%	24%	2.82 (1.36–5.84) *	1.19 (0.40–3.54)
Wore flea collars				
Ever	15%	4%	3.85 (0.89–16.67)	2.04 (0.38–10.86)
Saw living area sprayed/fogged with pesticides				
Ever	40%	24%	2.12 (1.02–4.40) *	0.91 (0.37–2.24)
≥7 days	23%	11%	2.48 (0.93–6.63)	1.17 (0.35–3.89)
Received one or more shots in arm in theater	75%	59%	2.52 (1.29–4.92) *	1.57 (0.70–3.51)
Received one or more shots in buttocks in theater	53%	37%	2.24 (1.16–4.32) *	1.23 (0.54–2.79)
Used pyridostigmine bromide (NAPP) pills				
Ever	82%	67%	2.25 (1.11–4.58) *	0.60 (0.23–1.56)
≥7 days	53%	37%	1.89 (0.98–3.64)	1.08 (0.49–2.38)
Contact with fresh CARC paint				
Ever	46%	13%	5.55 (2.25–13.65) **	2.13 (0.73–6.23)
≥7 days	27%	11%	2.96 (1.11–7.85) *	0.97 (0.30–3.09)
Experienced one or more mTBIs during deployment	37%	7%	8.33 (2.50–27.72) **	7.39 (1.64–33.28) *

Note: ^1^ Adjusted for use of skin pesticides ≥ 7 days, mTBI during deployment, age, rank, PTSD. * = significant association, *p* < 0.05; ** = significant association, *p* < 0.001. Abbreviations: GW = Gulf War; GWI = Gulf War illness, OR = odds ratio; CI = confidence interval; NAPP = nerve agent pyridostigmine pretreatment; CARC = chemical agent resistant coating; mTBI = mild traumatic brain injury; PTSD = posttraumatic stress disorder.

## Data Availability

Requests for GWIC data and samples can be made through online request from the Boston Biorepository and Integrative Network for Gulf War Illness (BBRAIN) website at http://sites.bu.edu/bbrain/data-request/ (accessed on 25 August 2021).

## Supplementary Materials

The following are available online at https://www.mdpi.com/article/10.3390/brainsci11091132/s1, Figure S1. GWIC Study Exclusionary Conditions.
