# Supplementary material for: Brain–Immune Interactions as the Basis of Gulf War Illness: Clinical Assessment and Deployment Profile of 1990–1991 Gulf War Veterans in the Gulf War Illness Consortium (GWIC) Multisite Case-Control Study
Source: Brain Sci. 2021 Aug 26;11(9):1132. doi: 10.3390/brainsci11091132 (PMC8467437; doi:10.3390/brainsci11091132)
Supplement: Supplementary file 1 [file brainsci-11-01132-s001.zip › brainsci-1325041-supplementary.pdf]

Supplemental Figure 1:
Gulf War Illness Consortium (GWIC) Study Exclusionary Conditions

Exclude if condition was ever diagnosed by a physician	- Multiple Sclerosis<br>- Lupus<br>- Rheumatoid Arthritis<br>- Stroke<br>- Parkinson's Disease<br>- ALS (Lou Gehrig's Disease)<br>- Alzheimer's Disease<br>- Bipolar Disorder<br>- Schizophrenia
Exclude if diagnosed condition has been present in the last 5 years	- Seizure disorder<br>- Heart disease (high blood pressure, high cholesterol not exclusionary)<br>- Kidney disease<br>- Liver disease<br>- Cancer (non-melanoma skin cancer not exclusionary)
Excluded if veteran was hospitalized for condition in the last 5 years	- Post-traumatic stress disorder (PTSD)<br>- Depression<br>- Alcohol or drug dependence
Specific Exclusions	- Diabetes: Exclude if blood sugar has not been well-controlled for the prior 2 years<br>- Chronic infectious disease: Exclude for any current chronic infectious disease lasting 6 months or longer<br>- Serious injury: exclude if veteran is currently recovering from serious injury<br>- Other diagnoses: Exclude any other reported current diagnosis lasting $\geq$ 6 months that could account for veterans' symptoms
